# Do financial incentives for delivering health promotion counselling work? Analysis of smoking cessation activities stimulated by the quality and outcomes framework

**DOI:** 10.1186/1471-2458-10-167

**Published:** 2010-03-26

**Authors:** Tim Coleman

**Affiliations:** 1 Reader in Primary Care, UK Centre for Tobacco Control Studies, Division of Primary Care, University of Nottingham, Medical School, Queen's Medical Centre, Nottingham, NG7 2UH, UK

## Abstract

**Background:**

A substantial fraction of UK general practitioners' salaries is now intended to reflect the quality of care provided. This performance-related pay system has probably improved aspects of primary health care but, using the observational data available, disentangling the impacts of different types of targets set within this unique payment system is challenging.

**Discussion:**

Financial incentives undoubtedly influence GPs' activities, however, those aimed at encouraging GPs' delivery of health promotion counselling may not always have the effects intended. There is strong, observational evidence that targets and incentives intended to increase smoking cessation counselling by GPs have merely increased their propensity to record this activity in patients' medical records. The limitations of using financial incentives to stimulate the delivery of counselling in primary care are discussed and a re-appraisal of their use within UK GPs' performance-related pay system is argued for.

**Summary:**

The utility of targets employed by the system for UK General Practitioners' performance related pay may be inappropriate for encouraging the delivery of health promotion counselling interventions. An evaluation of these targets is essential before they are further developed or added to.

## Background

Since 2004, UK general practitioners' (GPs') remuneration has been partially performance-related, governed by the Quality and Outcomes Framework (QOF) [1,2]. Through the QOF, GPs receive payments, representing up to 20% of their income [3], for compliance with targets (called 'indicators') set across the whole spectrum of clinical activity. General practices decide how payments are used and there is no requirement for these to be shared amongst the primary health care team; any diffusion of incentive payments to non-GP staff is controlled by those who run practices (usually GPs). Observational studies investigating this vast health system experiment show that the QOF has improved asthma and diabetes care [4,5]. Recorded care of cardiovascular disease is also better than before 2004 [4], but since the QOF there has been no acceleration in the rate of improvement [4,5]. Targets set in any incentives-based quality management system are of crucial importance and QOF indicators need to be regularly revised to ensure that they continue to stimulate further improvements in health care [6]. However, the validity of some QOF indicators for producing their intended effects is questionable. For example, GPs can comply with QOF targets for advising patients against smoking without actually delivering any useful or effective counselling. Whilst the QOF seems to have encouraged the use of effective diagnostic and drug interventions, it may not be an appropriate mechanism for promoting delivery of health promotion counseling and QOF indicators which rely on clinicians' records of having counseled or advised patients may be particularly prone to unintended consequences.

## Discussion

### QOF clinical indicators: validity

Valid QOF indicators would promote delivery of effective health interventions and, as most UK general practitioners work to the QOF, such targets could have massive potential for improving population health. For maximal impact, indicators would need to be aimed at people experiencing significant morbidity and to stimulate the use of interventions known to reduce this. As long as delivered interventions are always (or almost always) noted in patients' records, audits demonstrating QOF target achievement would also demonstrate improved clinical care. The QOF indicator for recording HBA1c levels in patients with diabetes (shown below) is a good example of a high-validity and, therefore, high-impact QOF indicator:

*'The percentage *of *patients with diabetes in whom the last HbA1c is *7.5* *or less (or equivalent test/reference range depending on local laboratory) in the previous *15 *months'*

*GPs are rewarded for having a specified percentage of patients with better than this level of control of diabetes

Target patients are clearly defined as those with diabetes mellitus, a high-morbidity disease with clear diagnostic criteria, in which maintaining blood glucose at lower levels reduces future harm [7]. It seems very likely that this indicator will encourage clinical activities to reduce diabetic patients' blood glucose levels. Additionally, as blood test results are almost always inserted automatically into primary care medical records, audited target compliance, which is based on medical records data, should directly reflect the quality of effective medical care for diabetic patients. This and other, similarly specific, indicators are probably responsible for the improved management of diabetes stimulated by the QOF [4,5]. However, not all QOF indicators are as well targeted and, in particular, those which rely on GPs' recording of counselling interventions may have effects other than those originally intended. QOF indicators related to smoking illustrate this phenomenon and are discussed below.

### QOF smoking indicators

Smoking is a major risk factor for many illnesses so, recording smoking status in medical records is necessary for good medical care. When this is noted prominently, health professionals are more likely to deliver or offer effective smoking cessation interventions [8]. like brief advice against smoking [9], nicotine replacement therapy (NRT) [10], bupropion [11] or varenicline [12]. Smoking status data in medical records is generally valid [13], as doctors are careful to accurately record this risk factor which is so relevant to their patients' health. Consequently, QOF indicators for the ascertainment and recording of smoking status could potentially have a positive impact on public health and, in the original 2004 QOF, 8% of quality payments could be obtained for smoking-related medical care. Smoking-orientated QOF indicators (examples below) were written to promote the ascertainment of patients' smoking status and the provision of smoking cessation advice to smokers and variations have been included in subsequent QOF revisions:

*'The smoking status of patients age *15 - 75 *is recorded for at least *55 *per cent of patients'*

'The percentage* of patients with coronary heart disease who smoke, whose notes contain a *record that smoking cessation advice has been offered within the last *15 *months'*

*GPs are rewarded for having recorded giving advice to a specified percentage of smokers

GPs' brief advice against smoking is essentially a simple, counselling intervention, delivered within patients' routine consultations, lasting no more than five minutes (often substantially less), during which doctors make clear that smoking is harmful and offer help with cessation [8].

Trials in many different health care settings have shown, unequivocally, that GPs' brief cessation advice against helps patients to stop smoking [8,14], so encouraging GPs to give and also record such advice is logical. However, GPs hold divergent opinions on what constitutes effective advice against smoking [15]; the 'advice' they give can vary from the briefest mention of smoking to detailed discussion of specific strategies to assist cessation attempts. Consequently, what GPs enter as 'brief advice' in patients' medical notes is likely to vary greatly and some may be prone to record even the slightest mention of smoking. Also, the indicator above rewards GPs for 'offering' rather than 'giving' advice, potentially allowing clinicians latitude for deciding whether or not to record that they have complied with this target. Below, research describing the impact of QOF smoking indicators on the delivery of primary care smoking cessation interventions is interpreted and discussed.

### QOF: impact on cessation interventions

**Figure 1:**
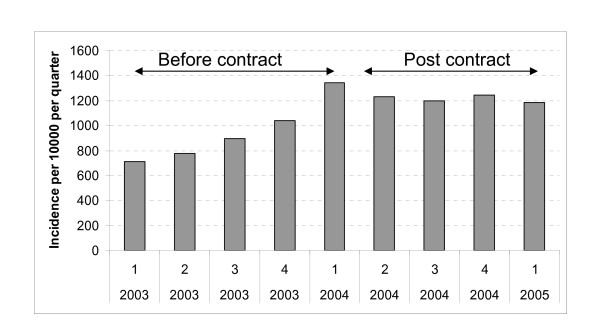
**Quarterly incidence of smoking status ascertainment in general practice patients measured in The Health Improvement Network database (2003-2005)**.

**Figure 2:**
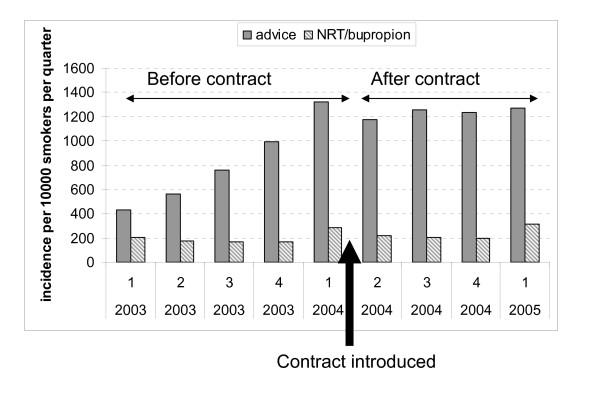
**Quarterly incidence of smoking cessation advice and prescriptions for nicotine replacement therapy and bupropion combined issued to smokers as recorded in The Health Improvement Network database (2003-2005)**.

Figures 1 and 2, based on data from over 4 million sets of primary care medical records contained in (The Health Improvement Network) THIN research database, show how the QOF initially affected documentation and recorded use of smoking cessation interventions in UK primary care [16]. Over the 12 months prior to the QOF being implemented, rates of smoking status ascertainment almost doubled and of cessation advice recorded in medical records nearly tripled and these substantial increases were sustained throughout following year. If these increases reflected real changes in clinical behaviour, then the impact of the QOF would be far higher than that of other established methods of influencing clinical behaviours like audit and feedback [17]. However, as no concomitant increase in the rate of prescribing nicotine addiction treatments occurred (Figure 2), these apparent increases in other smoking cessation intervention rates need to be questioned. If GPs really doubled their rates of enquiring about patients' smoking status and also tripled their advice-giving, a related but smaller increase in prescriptions for nicotine addiction treatments would be expected. At least some of the 'extra' discussions about smoking would have involved smokers who intended to try stopping soon, because 20% of smokers feel this way about their habit [18]. One would expect a proportion of such motivated smokers to either request or be offered and receive prescriptions for nicotine addiction treatments to assist impending cessation attempts. The expected increase in prescribing rates would probably be smaller than any increase in recorded advice-giving because pharmacotherapy for cessation is only appropriate for those smokers who are motivated enough to try to stopping. However, the absence of *any *change in prescribing, despite using a database with sufficient power to detect much smaller effects than those shown justifies a consideration of alternative explanations for the apparent increase in advice giving. Below, explanations for this discrepancy, based on previous research, are presented.

### More advice or better documentation?

GPs mention smoking in no more than 20% of smokers' consultations [18], selecting out those with smoking related problems and greater motivation to stop for advice [19]. When deciding who to advise, GPs try to avoid upsetting patients [20] and are more comfortable discussing smoking with smokers who want to stop [15]. GPs don't feel equipped with appropriate skills for advising patients who are undecided or not motivated to stop and usually curtail these consultations and also those in which smokers react negatively to the mention of smoking [15,21,22]. Most GPs' discussions about smoking are cursory, consisting of two questions, *"Do you smoke?" *and *"How many?" *andwhen advice is given, this usually involves simple exhortations to stop rather than more specific instructions [19]. Given the caution that GPs report in their approach to advising patients on smoking, a tripling in their rate of doing so would represent a very major change in their clinical behaviour.

The principal impact of the QOF could merely have been to increase GPs' recording of their advice in patients' medical records instead of actually altering GPs' rates of advising smokers. Such an increased propensity of GPs for recording their cessation advice would result in QOF compliance audits finding improved achievement of smoking-related QOF targets, without any or only minimal, increase in actual advice-giving. Prior to the QOF, GPs chose whether or not they documented advice against smoking in medical records and only a small minority of all advice given was documented [23]. GPs' low rates of rates of recording cessation advice appear relatively amenable to change as, for example, distributing questionnaires about smoking to patients sitting in doctors' waiting rooms, doubled GPs' advice-recording without their being any simultaneous increase in actual advice-giving [24]. The QOF now motivates GPs to record all advice against smoking in patients' medical records and it is plausible that the apparently QOF-related increases in 'advice-giving' are primarily due to increased rates of *documenting *advice given in medical records, so that QOF clinical indicators can be achieved and payments earned. In response to the QOF, some UK general practices have introduced major alterations to their organization of chronic disease care [25,26]; in comparison, increasing the completeness of documenting cessation advice would be relatively easy. Since the QOF was introduced, much chronic disease care has been delegated to practice nurses working with electronic templates to record data for QOF indicators [25]. Consequently, patients' smoking status is likely to have been primarily determined outside of GPs' consultations, reducing the potential for GPs' brief advice to be given or nicotine addiction treatments prescribed.

### Implications for smoking indicators

To have their intended, positive effects on unhealthy behaviours, health promotion targets must be very carefully phrased [27]; evidence suggests that the current QOF indicator for 'offering' smoking cessation advice is not valid and alternatives are needed. A QOF indicator which rewards prescribing of effective nicotine addiction treatments to motivated smokers would be a logical alternative. Electronic prescriptions are automatically documented in medical records and prescribing is an uncontroversial, uniform clinical activity; consequently, compliance with a prescribing target could not be influenced by GPs' propensities to record their clinical behaviours in medical records. Increased prescribing would also be likely to also result in more brief advice giving, because GPs would need to talk to many smokers to find those who were sufficiently motivated to benefit from pharmacotherapy.

### Implications for other QOF indicators

Evidence on smoking-orientated QOF indicators suggests that targets for delivering health promotion counselling may not result in advice or counselling actually being delivered. Additional file 1 lists 'counselling-type' indicators from the 2009 QOF; these reward GPs for giving information or advice on clinical issues such as alcohol intake, where the consensus about appropriate health promotion advice for individuals is less clear cut. One indicator rewards the provision of the following advice to those suffering from mental health conditions, "*routine health promotion and prevention advice appropriate to their [i.e. the patient's] age, gender and health status" *and quite what kind of advice giving this is intended to encourage is unclear. The QOF undoubtedly affects clinical behaviour and unambiguous indicators for clearly-defined, effective interventions (e.g. prescribing drugs or performing and recording diagnostic tests) are likely to affect these clinical behaviours. However, more caution is required when developing targets for less easily-defined, health promotion counselling interventions which clinicians document having given in medical records; it is very possible that these targets are not valid and don't stimulate intended health promotion counseling activity.

### Wider implications for health promotion

Despite the apparent success of the QOF in improving some aspects of clinical care, it has been criticised as being an obstacle to patient-centred medicine [28], for not sufficiently remunerating the extra effort required by practices working in deprived areas [29] and for encouraging neglect of clinical areas for which there are no clinical indicators [30]. The QOF's utility for encouraging effective health promotion counselling appears questionable and whether incentives payments are ever appropriate for this is open to debate. Health promotion payments were introduced into UK primary care in 1990 [31]; initially health promotion activity undertaken in specially-organised clinics, rather than during routine consultations was incentivised but soon discontinued on cost grounds; evaluation provided no evidence that, if continued, this health promotion approach would have been effective [32]. Subsequently, another primary care health promotion scheme involving target payments for recording cardiovascular risk factors and giving lifestyle advice to patients was introduced, but this too was quickly scrapped [33,34]. Like the QOF, this system rewarded GPs for counselling patients and also relied on the doctors themselves to record when they had done this. Evaluation suggested that doctors principally made administrative changes in their recording of patients' lifestyle data to enable incentives to be claimed, rather than substantially altering preventive activities [34]. Similarly, in an experimental study monitoring the introduction of an outcome based health promotion payment, in which GPs were remunerated for recording three month's abstinence from smoking by patients also encouraged administrative changes made to facilitate payment claims rather than meaningful clinical interventions against smoking [27].

## Summary

The utility of the QOF for increasing the use of effective, health promotion counselling interventions is questionable and the impact of current indicators aimed at achieving this should be rigorously-evaluated before additional similar targets are set. The revision process, led by the National Institute for Clinical Excellence [35], should aim to ensure that the QOF remains as relevant as possible to primary medical care and, therefore, needs to include an assessment of whether individual QOF indicators are having their desired effects, with those considered non-valid being modified or rejected.

## Abbreviations

UK: United Kingdom; GP: General Practitioner; QOF: Quality Outcomes Framework; HbA1c: Haemogloblin A1c; THIN: The Health Improvement Network (a research database containing primary care medical records).

## Competing interests

The author declares that, in the last 5 years, he has been paid for consultancy work by Johnson and Johnson and Pierre Fabre Laboratories (manufacturers of nicotine replacement therapy). However, this manuscript has not been discussed with any third parties.

## Authors' contributions

TC is the sole author of this paper because there have been no other contributors.

## Pre-publication history

The pre-publication history for this paper can be accessed here:

http://www.biomedcentral.com/1471-2458/10/167/prepub

## Supplementary Material

Additional file 1 **Clinical indicators for health promotion counselling-type interventions in the 2009 QOF**. This lists clinical indicators contained in the 2009 QOF which relate to 'counselling-type' interventions. Click here for file
