# Supplementary material for: Do financial incentives for delivering health promotion counselling work? Analysis of smoking cessation activities stimulated by the quality and outcomes framework
Source: BMC Public Health. 2010 Mar 26;10:167. doi: 10.1186/1471-2458-10-167 (PMC3091543; doi:10.1186/1471-2458-10-167)
Supplement: Additional file 1 — Clinical indicators for health promotion counselling-type interventions in the 2009 QOF. This lists clinical indicators contained in the 2009 QOF which relate to 'counselling-type' interventions. [file 1471-2458-10-167-S1.DOC]

**Indicators included in QOF revisions prior to 2009**

**PP 2**. The percentage of people diagnosed with hypertension diagnosed after 1 April 2009 who are given lifestyle advice in the last 15 months for: increasing physical activity, smoking cessation, safe alcohol consumption and healthy diet.

**MH 9**. The percentage of patients with schizophrenia, bipolar affective disorder and other psychoses with a review recorded in the preceding 15 months. In the review there should be evidence that the patient has been offered routine health promotion and prevention advice appropriate to their age, gender and health status

**Indicators introduced, for the first time, in the 2009 QOF**

**SH 3**: The percentage of women prescribed an oral or patch contraceptive method in the last year who have received information from the practice about long acting reversible methods of contraception in the previous 15 months.

**SH 4**: The percentage of women prescribed emergency hormonal contraception at least once in the year by the practice who have received information from the practice about long acting reversible methods of contraception at the time of, or within one month of, the prescription.

**Indicators under consideration by NICE in 2009**

‘The percentage of women with epilepsy under the age of 50 who are taking antiepileptic drugs who have a record of information and counselling about contraception, conception and pregnancy in the previous 15 months (unless not clinically necessary).’
